# Supplementary material for: Acetyl-CoA Carboxylase Inhibitor CP640.186 Increases Tubulin Acetylation and Impairs Thrombin-Induced Platelet Aggregation
Source: Int J Mol Sci. 2021 Dec 4;22(23):13129. doi: 10.3390/ijms222313129 (PMC8658010; doi:10.3390/ijms222313129)
Supplement: Supplementary file 1 [file ijms-22-13129-s001.zip › Octave et al, Supplementary corrected.pdf]

Figure S1

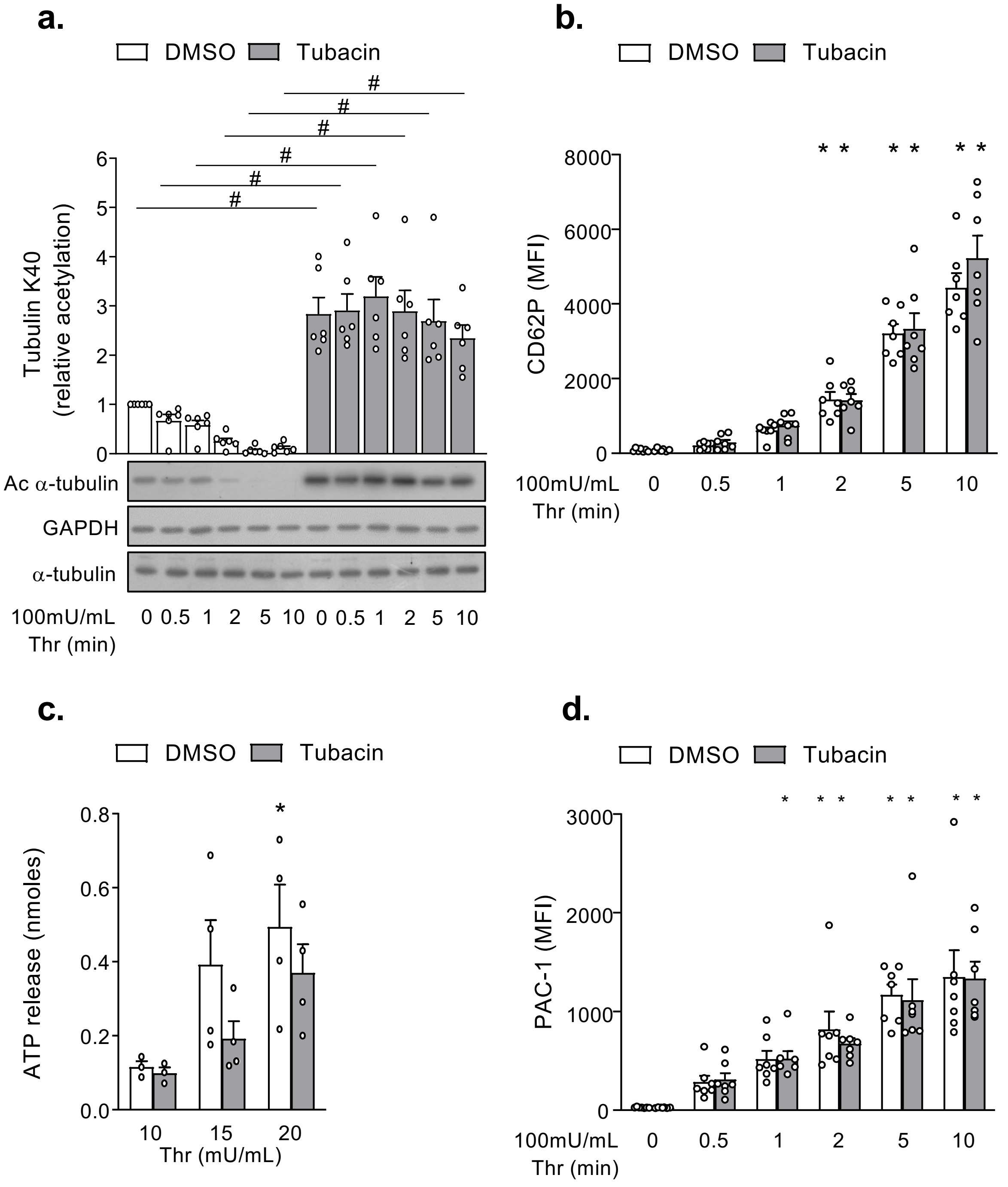

Figure S1. HDAC6 inhibition induced by tubacin increases α-tubulin acetylation level with no impact on granules secretion or αIIbβ3 activation upon thrombin stimulation.

(a-e) Washed human platelets were preincubated for 1 hour with DMSO or tubacin (10 μM) before being stimulated with thrombin (Thr). (a) Platelets were stimulated with 100mU/mL Thr at different time points. Whole platelet lysates were subjected to western blot and probed with acetyl α-tubulin (Ac α-tubulin), α-tubulin or gelsolin antibodies. Data are expressed as means ± SEM (n = 6). # p-value ≤ 0.0001 relative to DMSO condition. Data were compared using 2-way ANOVA. (b) P-selectin (CD62P) exposure was analyzed by flow cytometry in platelets stimulated with 100mU/mL Thr at different time points. Data are expressed as means ± SEM (n = 7). * p-value ≤ 0.05 relative to unstimulated condition. Data underwent 2-way ANOVA. (d) Dense granules secretion was assessed via addition of luciferase-luciferin reagent in platelets stimulated with different Thr concentrations. Data are expressed as means ± SEM (n = 4). Data were compared using 2-way ANOVA. (e) Platelets were stimulated with 100mU/mL thrombin (Thr) at different time points and αIIbβ3 activation (PAC-1) was detected by flow cytometry. Data are expressed as measn ± SEM (n = 7). * p-value ≤ 0.05 relative to unstimulated condition. Data were compared using 2-way ANOVA.

Figure S2

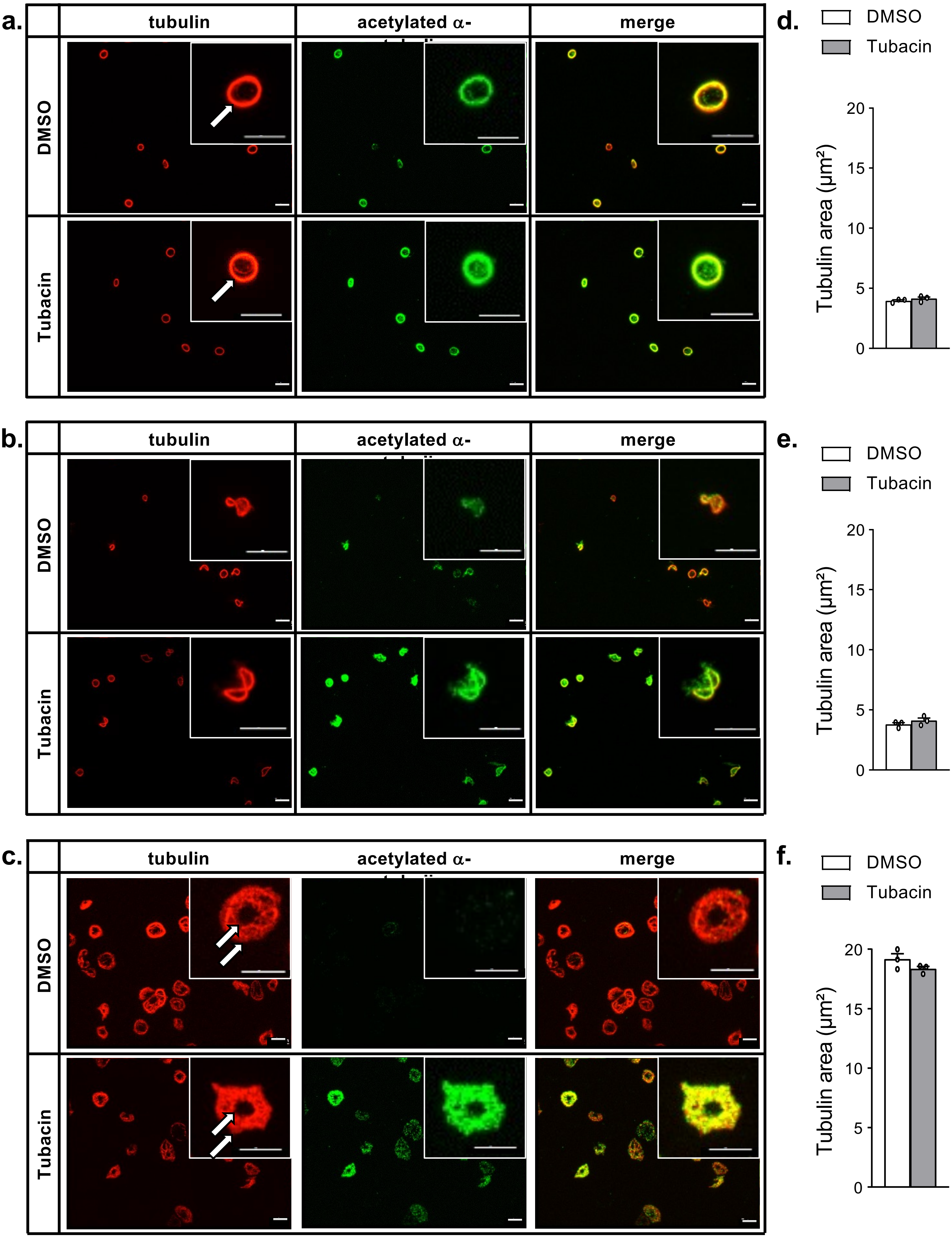

Figure S2. HDAC6 inhibition induced by tubacin does not affect microtubules organization.
(a-f) Washed human platelets were preincubated for 1 hour with DMSO or tubacin (10µM) before being added to fibrinogen-coated coverslips for 10 min. (a) Unstimulated platelets were fixed directly with 1% PFA and then added to coverslips. (b) Platelets were added to coverslips before being fixed with 1% PFA. (c) Platelets were stimulated with 100mU/mL thrombin and then added to coverslips before being fixed with 1% PFA. (a-c) Platelets were stained with tubulin (red) or acetyl α-tubulin (green) antibodies. Representative pictures are shown. Microtubule rings are indicated by white arrows. Scale bar, 5 µm. (d-f) Quantification of tubulin area. Data are expressed as means ± SEM (n = 3). Data were compared using unpaired t-test.

Figure S3

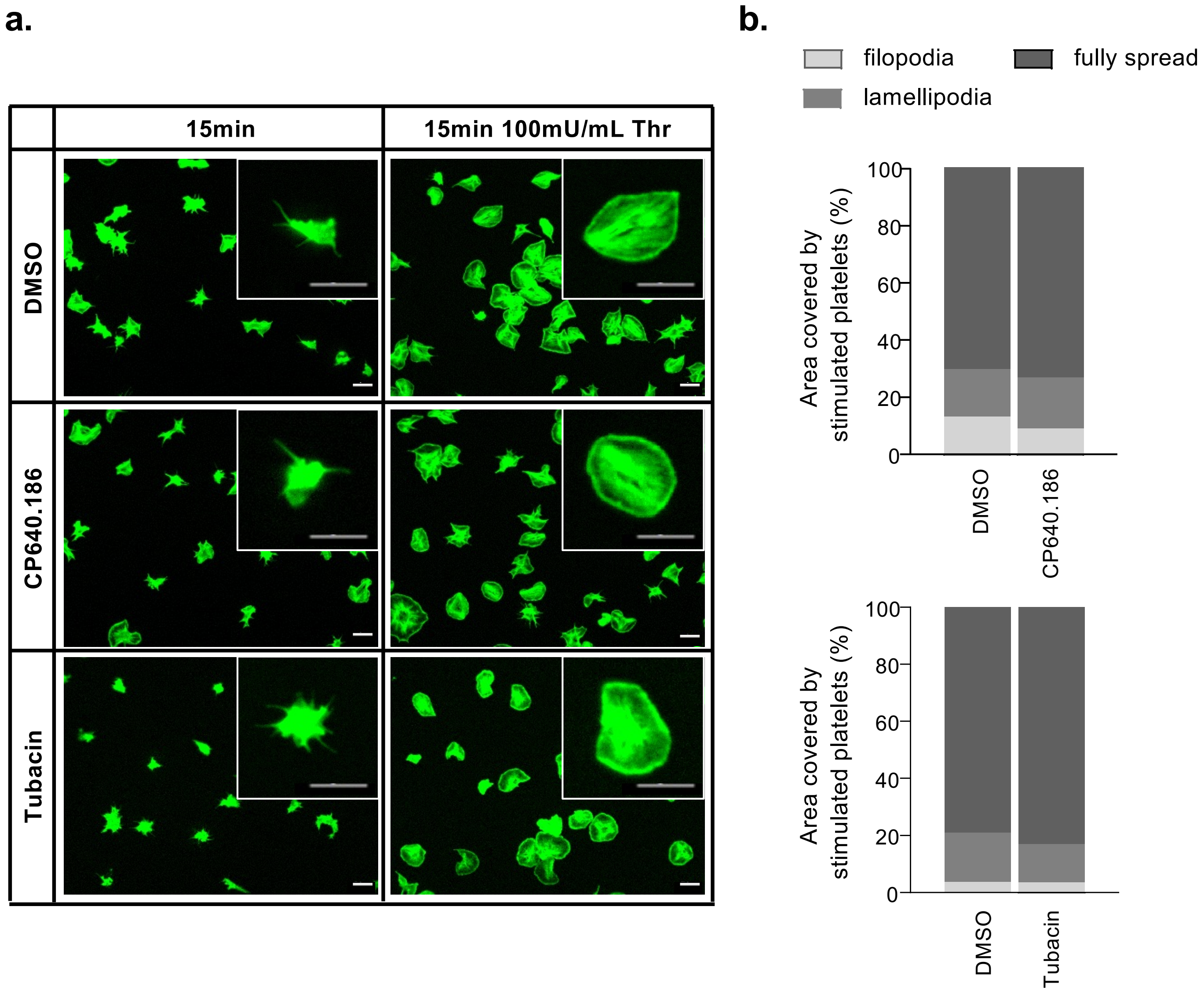

Figure S3. ACC or HDAC6 inhibition does not impact actin cytoskeleton remodeling.
(a-b) Washed human platelets were preincubated for 2 h with DMSO or CP640.186 (60 µM) or 1 h with tubacin (10µM) (a; left panel) without thrombin (Thr) or (a; right panel) after Thr stimulation (100mU/mL) for 15 minutes. Platelets were stained with phalloidin-FITC for 45 min. (a) Representative pictures are shown. Scale bar, 5 µm. (b) Quantification of area covered by stimulated platelets expressing filopodia, lamellipodia or being fully spread. Data underwent 2-way ANOVA.

Figure S4

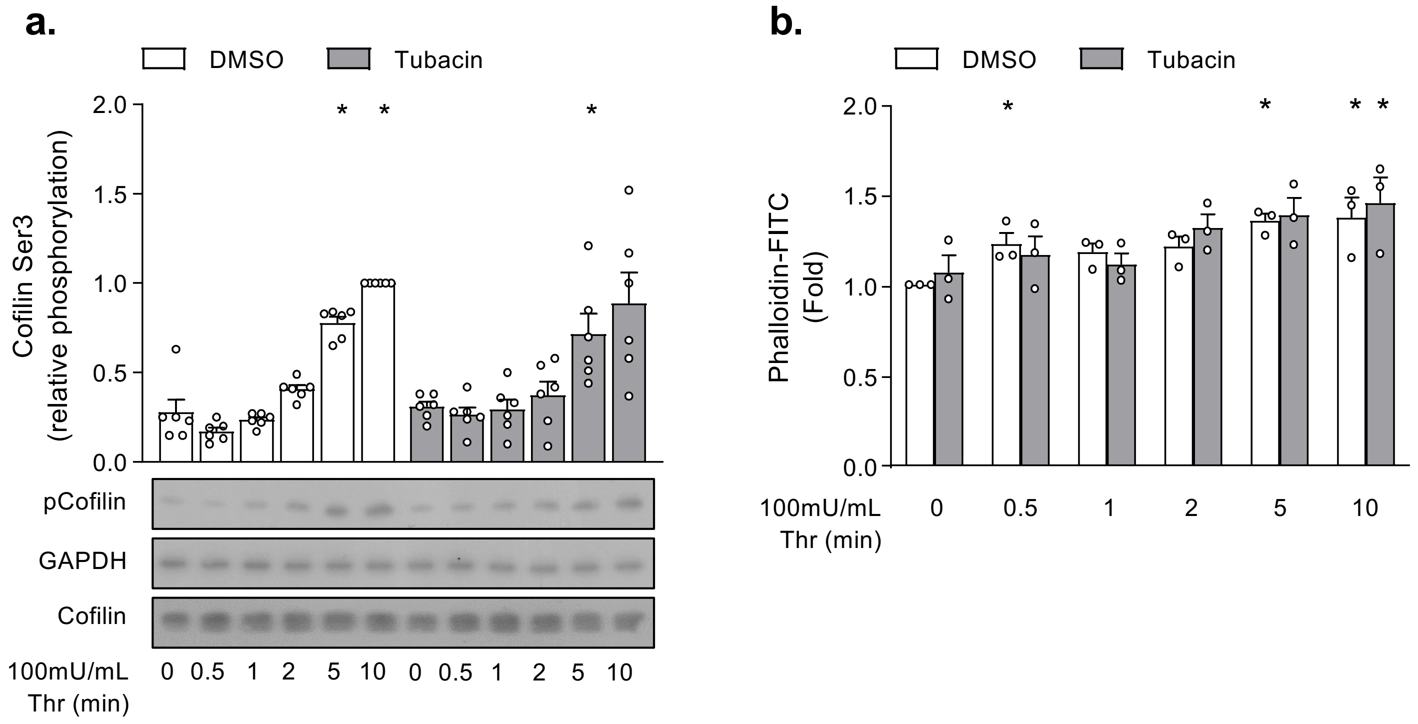

Figure S4. HDAC6 inhibition induced by tubacin reduces the activation of Rac1-PAK2 pathway in response to thrombin but does not affect actin polymerization.

(a-d) Washed human platelets were preincubated for 1 hour with DMSO or tubacin (10µM) before being stimulated with thrombin (Thr) (100mU/mL) at different time points. (a) Whole platelet lysates were subjected to western blot and probed with phosphoCofilin, Cofilin or GAPDH antibodies. Data are expressed as means ± SEM (n = 6). * p-value ≤ 0.05 relative to unstimulated condition. Data were analyzed using 2-way ANOVA. (b) Platelets were stained with FITC-conjugated phalloidin (10µM) for 1 hour. F-actin content was analyzed by flow cytometry. Data are expressed as means ± SEM (n = 3). * p-value ≤ 0.05 relative to unstimulated condition. Data were analyzed using 2-way ANOVA.

Figure S5

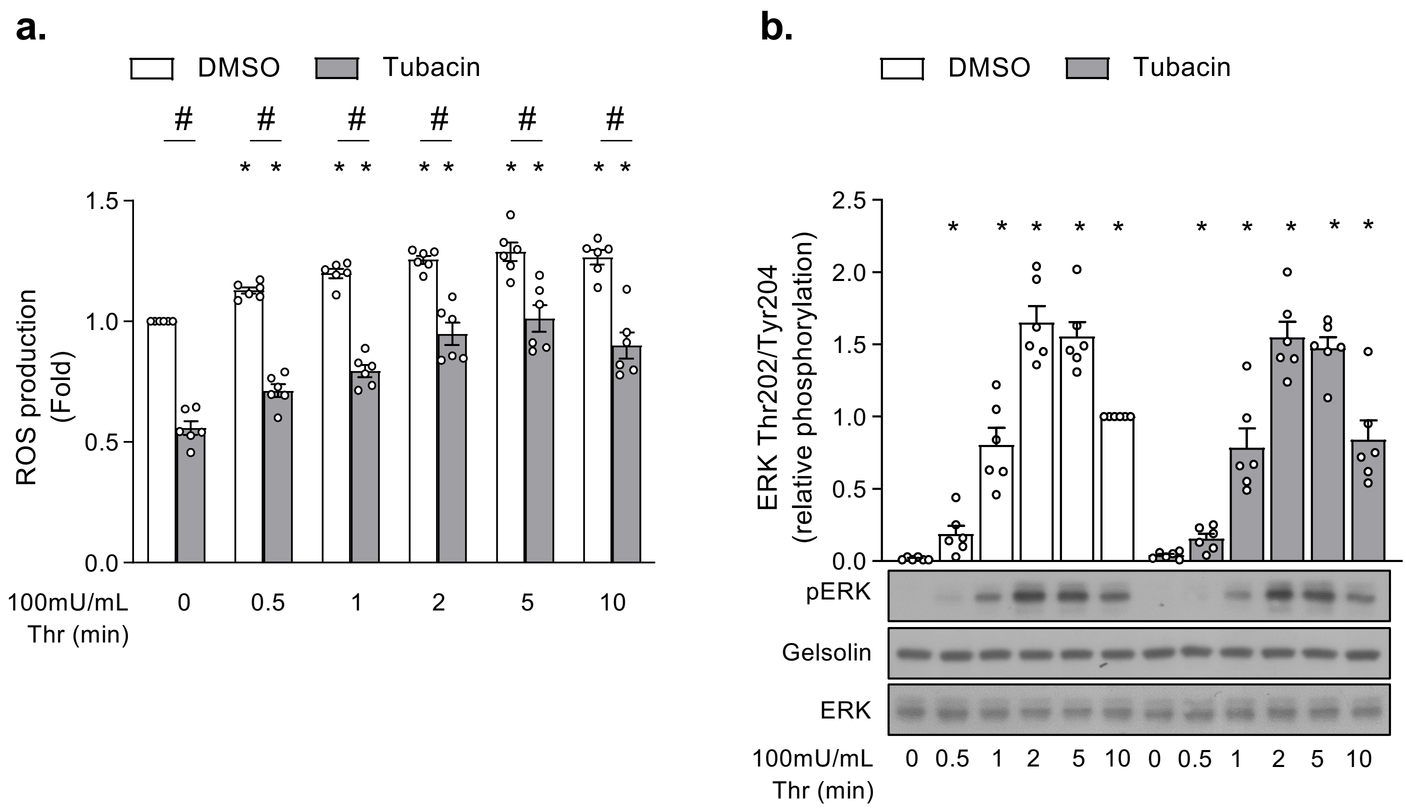

Figure S5. HDAC6 inhibition induced by tubacine decreases thrombin-induced reactive oxygen species (ROS) production but does not impact ERK phosphorylation.

(a-b) Washed human platelets were preincubated for 1 h with DMSO or tubacin (10µM) before being stimulated or not with thrombin (100mU/mL) at different time points. (a) Reactive oxygen species (ROS) were detected by flow cytometry using H_2DCFDA (10µM) probe. Data are expressed as means ± SEM (n = 6). * p-value ≤ 0.05 relative to unstimulated condition. # p-value ≤ 0.05 relative to DMSO condition. Data underwent 2-way ANOVA. (b) Whole platelet lysates were subjected to western blot and probed with phosphoERK, ERK or gelsolin antibodies. Data are expressed as means ± SEM (n = 6). * p-value ≤ 0.05 relative to unstimulated condition. Data underwent 2-way ANOVA..
